# Genetic Aspects of Gastric Cancer Instability

**DOI:** 10.1100/2012/761909

**Published:** 2012-04-19

**Authors:** Petra Hudler

**Affiliations:** Medical Centre for Molecular Biology, Institute of Biochemistry, Faculty of Medicine, University of Ljubljana, Vrazov trg 2, 1000 Ljubljana, Slovenia

## Abstract

Unravelling the molecular mechanisms underlying gastric carcinogenesis is one of the major challenges in cancer genomics. Gastric cancer is a very complex and heterogeneous disease, and although much has been learned about the different genetic changes that eventually lead to its development, the detailed mechanisms still remain unclear. Malignant transformation of gastric cells is the consequence of a multistep process involving different genetic and epigenetic changes in numerous genes in combination with host genetic background and environmental factors. The majority of gastric adenocarcinomas are characterized by genetic instability, either microsatellite instability (MSI) or chromosomal instability (CIN). It is believed that chromosome destabilizations occur early in tumour progression. This review summarizes the most common genetic alterations leading to instability in sporadic gastric cancers and its consequences.

## 1. Introduction

Gastric cancer remains a worldwide burden as one of the leading causes of cancer-related death in both sexes [1–3]. The late onset of clinical symptoms is the main reason that the disease is often diagnosed at an advanced stage, which limits available therapeutic approaches in more than 50% of cases [2–5]. Although extensive studies have been performed to identify genetic pathways and genes involved in the disease development and progression, the prognosis for patients with gastric cancer remains poor and little improvement of long-term survival has been achieved [6].

Adenocarcinoma is the major histological type of gastric cancer, accounting for 90% to 95% of all gastric malignancies. Based on the Lauren classification, which is mainly used in clinical setting, adenocarcinomas are divided into two distinct pathological entities, intestinal and diffuse types, which have different clinicopathological and prognostic features [3]. Intestinal type is associated with *Helicobacter pylori *infection, obesity, and certain dietary factors, such as high intake of salt, smoked meats, and food preserved with nitrites or nitrates, and is believed to arise through a long-term multistep progression from chronic gastritis to chronic atrophy to intestinal metaplasia to dysplasia [3, 7, 8]. Histologically it is well differentiated and occurs more commonly in older patients [7]. Diffuse type is poorly differentiated with infiltrating, noncohesive cells and is more frequent in younger patients [7, 9]. Studies showed that *Helicobacter pylori *infection also plays a role in the development of diffuse gastric cancer, through chronic inflammation, but without occurrence of intermediate steps, such as gastric atrophy and intestinal metaplasia [10].

During the last few decades the studies of gastric cancer showed that it results from the complex gene-environment interactions [9]. New high-throughput techniques have revealed its heterogeneous milieu with alterations of many genes, deregulation of signalling pathways, aberrant DNA methylation patterns, and chromosomal imbalances. Nevertheless, there is still no clear agreement on the genetic and epigenetic changes underlying the initiation and progression of gastric adenocarcinoma [9, 10]. Furthermore, it is evident that DNA polymorphisms along with individual's immune function and environmental factors contribute to the disease [10]. Even more, the biological characteristics of gastric tumours vary from case to case, and tumours from one individual are comprised of malignant cells showing different characteristics, growth preferences, and expression patterns [11]. Due to these facts and aggressive behaviour of most subtypes of gastric adenocarcinomas, it is difficult to choose the optimal therapeutic approach.

This paper is intended to focus on most common genetic aspects of genomic instability in gastric carcinogenesis and presents the current knowledge on some of the mechanisms and molecular pathways of malignant transformation.

## 2. Chromosomal Instability (CIN)

Chromosomal instability (CIN) is the most common type of genomic instability observed in solid tumours [5, 12]. CIN is characterized by gross chromosomal abnormalities, such as gain or loss of whole chromosomes (aneuploidy) and/or fractions of chromosomes (loss of heterozygosity (LOH), amplifications, and translocations) [13]. These alterations could also affect the expression of oncogenes, tumour suppressor genes, and other genes, such as genes implicated in digestion, DNA repair genes (genome stability genes), growth regulators, and cell cycle checkpoint control genes, and so CIN has been detected as the most common feature of sporadic gastric cancers and has been reported in up to 84% of gastrointestinal tumours [5, 14]. Numerous DNA copy number variations have been reported, and subgroups with different patterns of DNA copy number alterations have been recognized, which have been associated with age, prognosis, lymph node status, and metastasis [9, 11, 15–18]. Buffart et al. explored comparative genomic hybridisation (CGH) profiles of gastric adenocarcinomas in young and old patients [19]. They found out that chromosome regions 11q23.3 and 19p13.3 contributed most to age-related differences in tumour profiles and that tumours of younger patients showed gains in chromosomal regions 6p21, 9p34, 11p15, 11q23, 17p13, 19p13, and 22q13, whereas in the majority of older patients normal copy status was observed. These differences in genomic profiles likely reflect different pathogenic mechanisms of the disease. Varis et al. similarly observed that the most frequent cytogenetic aberrations were gains seen at 17q, 19q, and 20q in younger patients [20]. Furthermore, in another study, Buffart et al. showed that gastric cancers from South African and UK patients differ in genetic instability patterns, indicating possible different biological mechanisms of carcinogenesis in patients from different geographical origin [12]. Their results also revealed that South African patients showed significantly more microsatellite instable gastric cancers compared to Western European patients. Tsukamoto et al. also observed high frequencies of DNA copy number aberrations; for example, the 20q13 chromosome gain was detected in 97% of cases [21]. They used laser microdissection method to isolate tumour cells; therefore, their samples contained less cells from tumour microenvironment. They also identified 114 upregulated candidate genes located in regions of amplification and 11 downregulated genes located in regions of deletion.

LOH is also a marker of chromosomal instability and might indicate a second inactivation hit of cancer suppressor genes. Several LOH studies demonstrated that the extent of chromosomal loss appeared to be of prognostic significance [22–24]. We and other researches showed that two distinct subtypes, high-level LOH (named LOH-H) and low-level LOH (named LOH-L), could be correlated with intestinal or mixed and diffuse growth patterns, respectively [23, 25]. LOH has been shown to relate to cancer progression, where a transition from LOH-L to LOH-H is thought to reflect an increase in chromosomal instability during tumour advancement. We also found that the highest frequency of LOH was at APC locus (36%), followed by TP53-1 (33%), nm23 (33%), TP53-2 (24%), and RB (24%) [23]. Recently, Karaman et al. found significant correlation between prevalence of 17p (TP53) LOH in gastric precancerous lesions, indicating that loss of TP53 could be an early event in gastric carcinogenesis [26]. The highest LOH frequencies have been identified at 1p, 2q, 3p, 4p, 5q, 6p, 7p, 7q, 8p, 9p, 11q, 12q, 13q, 14q, 17p, 18q, 21q, and 22q chromosome regions in gastric carcinomas [9, 27]. The main consequence of LOH is loss of genes, such as tumour suppressors, cell cycle regulators, DNA repair genes, and other genes implicated in the maintenance of cell cycle and/or integrity of DNA.

The genetic mechanisms leading to CIN are largely unknown. It has been suggested that chromosome segregation defects, defective DNA damage response, aberrations in cell cycle regulators, and telomere dysfunction could lead to numerical and structural chromosome alterations [28, 29]. Furthermore, carcinogens such as *Helicobacter pylori *infection, tobacco, nitrates, and nitrites have an important impact on chromosomal stability in genetically susceptible individuals [4]. Chemical and physical carcinogens induce the malignant transformation by altering either chromosomes or the spindle apparatus by disrupting microtubules [30–32]. Certain carcinogens, such as tobacco, also reduce antioxidant capabilities and enhance lipid peroxidation and oxidant-mediated tissue damage, thus increasing the risk of gastric cancer [33].

### 2.1. Chromosome Segregation Dysfunction Leading to Chromosome Instability

Segregation is one of the fundamental processes in cells, which are rapidly dividing, such as gastric epithelial cells. Therefore, if regulation mechanisms, governing this process are damaged, the cells might proceed through cytokinesis with DNA or spindle errors and could inherit unrepaired mutations or gain an abnormal number of chromosomes [34]. However, the molecular defects underlying CIN and whether it is a cause or consequence of tumour phenotype are not completely clear. At least three possible mechanisms for CIN development have been suggested: aberrant expression, mutations and/or polymorphisms in mitotic genes, implicated in chromosome segregation, or the activity of carcinogens on susceptible genetic background of individuals [35, 36]. Several studies presented evidence that altered expression of mitotic genes could affect chromosome segregation. For example, Grabsch et al. observed overexpression of BUB1 protein in gastric cancers, which was significantly higher in tissues of patients with diffuse-type adenocarcinomas [14]. However, their study did not reveal any association between BUB1 protein expression level and DNA ploidy status of examined tumour types. BUB1 along with BUBR1, Aurora kinase B (AURKB), TTK, and other proteins is implicated in controlling the spindle assembly checkpoint. Ando et al., on the other hand, found a significant high correlation between BUBR1 overexpression and DNA aneuploidy [37]. Interestingly, in another study investigating the expression status of BUBR1 and AURKB, the authors concluded that overexpression of BubR1 and AURKB is associated with a low risk of gastric cancer progression [38]. MAD1 and MAD2 are also important regulators of cell cycle progression, involved in alignment of chromosomes at the metaphase plate. Studies suggested that MAD1 and MAD2 could be tumour suppressor genes, based on their reduced expression in gastric carcinomas [39, 40]. Aurora kinase A (AURKA or STK15) located at 20q13, a region that is frequently amplified in gastric cancer, has been found overexpressed in stomach adenocarcinomas [41]. AURKA is a cell-cycle-regulated kinase that appears to be involved in microtubule formation and/or stabilization at the spindle pole during chromosome segregation, thus ensuring equal partition of replicated chromosomes to daughter cells. Functional analysis of upregulated AURKA gene revealed a possible novel oncogenic pathway, involved in gastric carcinogenesis. AURKA overexpression led to a significant increase in mRNA levels of several direct targets of the *β*-catenin/TCF transcription complex (cyclin D1, c-MYC, c-MYC binding protein, CLDN1, FGF18, and VEGF) [42]. It was shown that AURKA overexpression overrides the mitotic spindle checkpoint and leads to incomplete cytokinesis, multinucleation, and multipolar spindles [43, 44]. Other important regulators of cell cycle progression, such as CCNB1, CCNE1, PTTG1, and PLK, have also been found overexpressed in gastric carcinomas and associated with poor prognosis [45–48].

Studies on several animal species and humans showed that certain genetic alterations and mutations in segregation genes might cause an increased incidence of a particular tumour type [49, 50]. However, these and several other studies explored overexpression and/or mutations of these genes, which could already be the consequence of CIN. Therefore, it has been proposed that minor alterations in mitotic genes could contribute to the onset of cancer [51]. High-throughput genotyping is suggesting that subtle variations, such as polymorphisms or nonlethal mutations, might induce CIN and aneuploidy. This hypothesis of low-penetrance allelic variants or risk alleles is further supported by the late onset of nonheritable cancers, whereas dominant mutations in oncogenes and tumour suppressors usually induce the disease early in life [31, 51]. Genetic variants in mitotic genes in combination with environmental factors could modulate mitotic pathways, thus causing slow accumulation of chromosomal aberrations in replicating epithelial cells. The key turnaround in this process is the impairment of critical oncogenes and tumour suppressors which enable uncontrolled proliferation and growth of cells. The search for “driving” alterations in these low-penetrance genes has begun only recently, and further investigations on larger cohorts are needed to establish the biological basis for the role of risk alleles of mitotic genes and their involvement in gastric carcinogenesis.

### 2.2. Defective DNA Damage Response

Cells of gastric mucosa are constantly exposed to different environmental and intracellular mutagens, such as naturally occurring reactive oxygen and nitric oxide species (RONS), *Helicobacter pylori *infection (discussed below), natrium (salt), nitrates, nitrites, and other environmental contaminants in water and food. These mutagens have been shown to induce DNA damage through different mechanisms [4, 6]. The main DNA repair pathways, responsible for maintaining the integrity of gastric cell genome, are base excision repair (BER) for repair of endogenous/oxidative damage, nucleotide excision repair (NER) for adducts, mismatch repair (MMR) for single-nucleotide mismatches and DNA polymerase slippage, and recombination and/or DNA damage response (DDR) for repairing double-strand breaks [52, 53]. Failure of these pathways can lead to chromosomal instability and/or to accumulation of genetic alterations, which favour neoplastic transformation. Guo et al. studied the phosphoproteome and transcriptome of gastric cancer cell lines and tissues of gastric cancer patients, patients with gastritis, and normal subjects. Using a combined approach with LC-MS/MS-based and protein antibody arrays, they established that DDR pathway appears overrepresented in investigated specimens (overexpressed MRE11, RAD1, RAD9, CHK2, and p53 proteins). Several studies also revealed differentially expressed mRNA of genes, involved in different DNA repair mechanism, such as ATM (involved in BER), HMGB1 (involved in BER), RAD23B (involved in NER), UBE2V2, MUS81 (involved in resolving Holliday junctions), REV3L (implicated in translesion replication after DNA damage), TP53, hHR23A (involved in NER), DDB1 (involved in NER), and XRCC1 (involved in repair of single-strand breaks), MUTYH (involved in BER) [45, 54–58].

It is also very likely that inherited polymorphisms in DNA repair genes could influence the host ability to detect and repair DNA damage in the presence of the permissive microenvironment [53]. Palli et al. investigated the effect of selected polymorphism in DNA repair genes alone and in combination with polymorphisms in GST genes, involved in ROS detoxification, on gastric cancer risk [53]. They found a borderline association between XPC-PAT+/+ genotype and increased risk for the development of gastric cancer. However, when they explored gene-gene interactions, they observed significant interactions between polymorphisms in DNA repair genes and also in interactions between DNA repair polymorphisms in combination with GST polymorphisms. For instance, the interactions between DNA repair gene polymorphisms APE1-D148E (BER) and XPA-23G>A (NER), and between XPC-PAT (NER) and XPA-23G>A (NER), showed a high association. A significant association was also confirmed between APE1-D148E (BER) and GSTT1 and between APE1-D148E (BER) and GSTM1-GSTT1 double-null genotype. The authors did not find significant associations between selected polymorphisms in other DNA repair genes, such as OGG1 (BER), XRCC1 (BER), XPD (NER); XRCC3 (NER), ERCC1 (NER), MGMT (NER). However, the homozygous carriers of W allele of this XRCC1 polymorphism (R194W) were highly associated with increased gastric cancer risk in Chinese and Japanese populations [59–61]. Several other polymorphisms in different DNA repair genes have been extensively studied with conflicting results regarding their association with cancer risk in different populations [62–67]. It is believed that inconsistency of these reports could underlie differences in ethnicity, lifestyle, and disease prevalence [53]. Furthermore, only a few studies addressed the additive effect of these polymorphisms alone or in combination with polymorphisms in other genes on gastric cancer risk. There is also a lack of studies on their interactions and biological behaviour in combination with different environmental triggers. Altered function of these low-penetrance genes due to polymorphisms may affect gene-environment and gene-gene interactions, thus favouring slow neoplastic transformation, which is characteristic for sporadic gastric cancers. In addition to affecting gastric cancer risk, inherited polymorphisms are also associated with efficacy and toxicity of chemotherapeutics [68].

### 2.3. Helicobacter-pylori-Induced Chromosome Instability

It has been shown that *Helicobacter pylori* triggers DNA double-strand breaks and DNA damage response (DDR) in cultured gastric adenocarcinoma and murine primary gastric epithelial cells in a process that probably requires adhesion of viable bacteria through interaction of BabA adhesin and host's Lewis epitopes [69]. In addition, Toller et al. also assessed the ability of cultured cells to repair the fragmented DNA. They observed that after short-term infection of gastric cells the cells retain their ability to repair double-strand breaks, while prolonged infection leads to elevated cell lethality, probably through saturation of repair mechanisms. The overloaded repair machinery (or inefficiency of repair proteins due to other mutations and/or polymorphisms) could cause inefficient and mutagenic double-strand break repair, thus inducing carcinogenic transformation. Furthermore, persistent infection with *Helicobacter pylori* initiates chronic inflammation, which induces increased tissue turn-over, increased rate of mutagenesis, oxidative-stress-related changes in proteome, downregulation of base excision and mismatch repair mechanisms, and genetic instability, and modulates apoptosis through the generation of reactive oxygen and nitrogen species (RONS) [10, 70–74]. Specifically, generation of RONS, produced by recruited immune cells to the site of inflammation, has been associated with oxidative DNA damage, DNA single-strand breaks, crosslinking of DNA, direct mutation of TP53 gene, protein damage by nitrosylation, inhibition of apoptosis by nitrosylation of caspases, and promotion of angiogenesis [75, 76]. Furthermore, host genetic background also plays an important role in *Helicobacter-pylori-*induced gastric cancer. Increased risk of cancer development in individuals infected with this bacteria has been associated with polymorphisms in genes implicated in gastric acid secretion, immune response, adhesion to epithelial cells, and other biological functions [4]. For example, it has been reported that a combination of polymorphisms within proinflammatory genes IL-1*β*, IL-1RA, TNF*α*, and IL-10 conferred greater risk for gastric cancer development in combination with *Helicobacter pylori *infection [77, 78]. Although the exact mechanisms of *Helicobacter-pylori*-induced carcinogenesis are not yet elucidated, it is believed that the combination of microenvironment, virulent microorganism, persistent inflammation response, and a genetically susceptible host drives the process of gastric cell transformation [79]. 

## 3. Microsatellite Instability (MSI)

Another type of genomic instability, commonly recognized in gastric cancer, is microsatellite instability (MSI). Studies showed that MSI is characteristic for hereditary type of gastric cancer, developed in the context of the Lynch syndrome, and a smaller subset of sporadic cancers ranging from 25% to 50% [5]. Patients with MSI phenotype exhibit a high frequency of replication errors resulting in insertions/deletions of nucleotides within microsatellite repeats in tumour tissues [5]. These errors are detected and repaired by a complex of mismatch repair (MMR) proteins [78]. Inactivation or deficiency of one or more MMR genes, particularly *MLH1* or *MSH2*, induces development of MSI phenotype, which often leads to additional genetic changes, namely inactivation of tumour suppressor genes and LOH [80, 81]. Impairment of MMR can occur (1) by mutational inactivation of one or two MMR genes or (2) by epigenetic inactivation of MMR genes (CpG island methylator pathway, CIMP). In gastric cancer, functional inactivation of MMR is mainly caused by latter. Epigenetic hypermethylation of *MLH1 *promoter has been found to be responsible for the development of more than 50% MSI-H-positive gastric cancers, whereas mutations in *MLH1* and *MSH2* are being reported in 12–15% of gastric cancers exhibiting MSI-H phenotype [82–85]. Genomes of gastric cancers exhibiting MSI are characterized by the presence of multiple frameshift mutations in many genes at variable frequencies [2]. Genes that were frequently found to be altered as a consequence of impaired MMR are implicated in cell cycle regulation and apoptosis (TGF*β* RII, IGFIIR, TCF4, RIZ, BAX, CASPASE5, FAS, BCL10, and APAF1) or are involved in genomic integrity maintenance (MSH6, MSH3, MED1, RAD50, BLM, ATR, and MRE11) [5, 86]. The alterations in these genes further promote genetic instability and enhance the development of malignant phenotype.

## 4. Deregulation of Signalling Pathways

**Figure 1:**
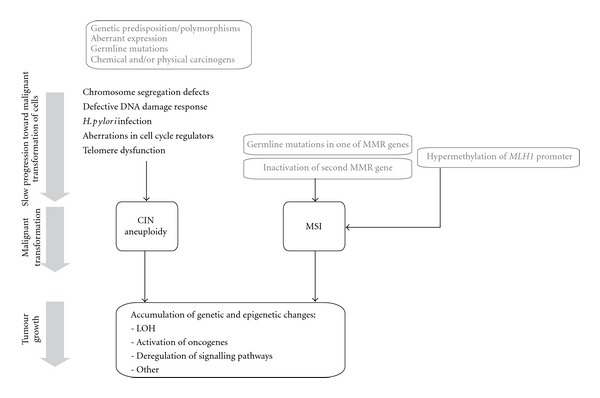
The pathogenic mechanisms inducing CIN or MSI lead to gastric cancer development.

The pathogenic mechanisms inducing genome instability could be responsible for deregulation of signalling pathways implicated in gastric homeostasis (Figure 1). The main consequences of genomic destabilization are aneuploidy, gains and losses of chromosome regions, resulting in amplification of oncogenes and/or LOH; translocations, inversions, and accumulation of point mutations, insertions, and deletions in coding sequences, regulatory sequences, and noncoding sequences, implicated in processing of mRNA transcripts. All these changes affect normal biological behaviour of cells, and certain combinations of changes induce the development and establishment of proliferating, invasive cell populations, which form the tumour mass. The malignant cells inherit the driving genetic mutations and/or polymorphisms implicated in instability generation; therefore, the process of genomic destabilization continues, explaining the heterogeneity of gastric tumours.

With the development of new high-throughput methods, it became evident that aberrations of certain signalling pathways could be important in gastric carcinogenesis. Furthermore, a multitude of genetic changes found in gastric cancers implies that many of the changes affecting the pathways could be individual; for example, patients could have different genes affected within the pathway, but resulting in the same final effect-deregulated pathway driving and sustaining neoplastic cells. Some of these pathways were initially discovered to be active during embryogenesis, but recent research showed that they are also involved in regeneration process of gastric mucosa [87]. As expected, global expression analyses showed aberrant expression of many genes, which differ among individuals, but are implicated in the same signalling networks [45, 87–91]. Furthermore, there is a complex interplay between environmental factors and these pathways that remains to be elucidated. The most studied pathways that likely contribute to gastric pathogenesis are Wnt/beta-catenin, extracellular signal-regulated MAPK, Hedgehog, Notch, COX2/PGE2, NF-*κ*B, TGF-*β*/BMP pathways, and tyrosine kinase signalling [45, 92–113]. The molecular mechanisms underlying the oncogenic signalling of these pathways have been extensively reviewed before [10, 91, 114]. Recent studies revealed that deregulation of pathways implicated in systemic inflammatory response, for example, IL-11/gp130/STAT1/STAT3, could also encourage malignant transformation [115–117]. In a gp130 (Y757F/Y757F) mouse model of gastric cancer, it was demonstrated that mutated gp130 activates STAT3, resulting in downstream activation of inhibitory protein SMAD7, which blocks stromal TGF-*β* signalling, thus suppressing the cytostatic effect of surrounding stromal cells on tumour cell proliferation. Stat3 could also enhance oncogenic signalling by inducing IL-11 expression, which activates mutated gp130, further promoting the signalling cycle and proliferation of aberrant cells. [115]. Additionally, gp130 also activates JAK/STAT and SHP-2/ras/MAPK/ERK1-2/AP-1 signalling cascades, resulting in the suppression of TFF1 tumour suppressor gene, and concomitant STAT3 activation results in mucosal atrophy, metaplasia, cellular proliferation, dysplasia, and submucosal invasion [118]. Another well-characterized genetic model of gastric carcinogenesis includes inactivation of RUNX3, a downstream target of TGB-*β* signalling pathway, resulting in the suppression of the apoptosis in gastric tumour cells [119]. Additionally, it should be noted that many of these pathways converge and that individually different combinations of aberrations in genes implicated in these signalling networks encourage or deter cancer development. Defining which pathways and how they are affected in individual patients could be of importance for the diagnosis and for determining the potential targets for cancer treatment.

## 5. Conclusion

Several other pathways leading to chromosomal instability in gastric cancers exist, which were not discussed, for example, telomere shortening, telomerase activation, relaxation of cell-cycle checkpoints, incomplete chromosomal replication, and so forth [29, 120]. The complexity and heterogeneity of gastric tumours provide evidence that gastric cancer is the consequence of a multistep process involving different genetic and epigenetic changes in numerous genes in combination with host genetic background and environmental factors. The number of altered genes implicated in gastric carcinogenesis is immense, and the importance of these changes is not clear [10]. Genetic instability plays an important role in neoplastic initiation and progression, and according to research, is an early event in gastric carcinogenesis. However, exact driving factors responsible for genome destabilization have not yet been found. In addition, complex gene-gene interactions, gene-environment interactions, lifestyle, and ethnic background further complicate the elucidation of gastric pathogenesis. Complete genotyping of gastric cancer patients could offer a better insight in genetic polymorphisms and their interactions, thus enabling the isolation of populations with increased risk for the disease development. There is also a need to develop algorithms that could explain all these changes found in individuals and their interactions. Furthermore, it is necessary to perform large studies and meta-analyses to define panels of biomarkers for determination of susceptibility, for diagnosis, prognosis, and treatment.

## Abbreviations

BER: Base excision repair
CGH: Comparative genomic hybridization
CIMP: CpG Island methylator pathway
CIN: Chromosomal instability
CNV: DNA Copy number variation
DDR: DNA damage response
LOH: Loss of heterozygosity
MMR: Mismatch repair
MSI: Microsatellite instability
NER: Nucleotide excision repair
RONS: Reactive oxygen and nitrogen species.
